# Focal cerebral ischemia in the TNFalpha-transgenic rat

**DOI:** 10.1186/1742-2094-5-47

**Published:** 2008-10-23

**Authors:** L Creed Pettigrew, Mark S Kindy, Stephen Scheff, Joe E Springer, Richard J Kryscio, Yizhao Li, David S Grass

**Affiliations:** 1 Sanders-Brown Center on Aging, University of Kentucky, Lexington, Kentucky, USA; 2 Department of Neurology, University of Kentucky, Lexington, Kentucky, USA; 3 Veterans Administration (VA) Medical Center, Lexington, Kentucky, USA; 4 Department of Neuroscience, Medical University of South Carolina, Charleston, South Carolina, USA; 5 Ralph H. Johnson VA Medical Center, Charleston, South Carolina, USA; 6 Department of Physical Medicine & Rehabilitation, University of Kentucky, Lexington, Kentucky, USA; 7 Department of Statistics and School of Public Health, University of Kentucky, Lexington, Kentucky, USA; 8 Jinan Great Wall Hospital, Jinan, Shandong, PR China; 9 Xenogen Biosciences, Cranbury, New Jersey, USA

## Abstract

**Background:**

To determine if chronic elevation of the inflammatory cytokine, tumor necrosis factor-α (TNFα), will affect infarct volume or cortical perfusion after focal cerebral ischemia.

**Methods:**

Transgenic (TNFα-Tg) rats overexpressing the murine TNFα gene in brain were prepared by injection of mouse DNA into rat oocytes. Brain levels of TNFα mRNA and protein were measured and compared between TNFα-Tg and non-transgenic (non-Tg) littermates. Mean infarct volume was calculated 24 hours or 7 days after one hour of reversible middle cerebral artery occlusion (MCAO). Cortical perfusion was monitored by laser-Doppler flowmetry (LDF) during MCAO. Cortical vascular density was quantified by stereology. Post-ischemic cell death was assessed by immunohistochemistry and regional measurement of caspase-3 activity or DNA fragmentation. Unpaired *t *tests or analysis of variance with post hoc tests were used for comparison of group means.

**Results:**

In TNFα-Tg rat brain, the aggregate mouse and rat TNFα mRNA level was fourfold higher than in non-Tg littermates and the corresponding TNFα protein level was increased fivefold (p ≤ 0.01). Infarct volume was greater in TNFα-Tg rats than in non-Tg controls at 24 hours (p ≤ 0.05) and 7 days (p ≤ 0.01). Within the first 10 minutes of MCAO, cortical perfusion measured by LDF was reduced in TNFα-Tg rats (p ≤ 0.05). However, regional vascular density was equivalent between TNFα-Tg and non-Tg animals (p = NS). Neural cellular apoptosis was increased in transgenic animals as shown by elevated caspase-3 activity (p ≤ 0.05) and DNA fragmentation (p ≤ 0.001) at 24 hours.

**Conclusion:**

Chronic elevation of TNFα protein in brain increases susceptibility to ischemic injury but has no effect on vascular density. TNFα-Tg animals are more susceptible to apoptotic cell death after MCAO than are non-Tg animals. We conclude that the TNFα-Tg rat is a valuable new tool for the study of cytokine-mediated ischemic brain injury.

## Background

Tumor necrosis factor-α (TNFα) is a pleiotropic cytokine suspected to enhance or deter cellular survival through activation of receptor-mediated signal transduction. When present in supra-physiological levels after injury, it is known to modulate neural cell loss in cerebral ischemia [1], intracerebral hemorrhage [2], chronic cerebral oligemia [3], and trauma [4]. The level of TNFα in human brain becomes elevated after cerebral infarction [5] and appears sequentially in the infarct core and peri-infarct areas before expression in tissue within the unaffected hemisphere [6]. Elevated levels of TNFα have been observed consistently in serum [7-9] and in cerebrospinal fluid [8,9] after acute ischemic stroke. In animal models of cerebral ischemia, high levels of TNFα have been found after global [10,11] and focal [12] ischemic injury. Several investigators reported reduction of infarct volume through inhibition of TNFα [13-15], although Nawashiro and colleagues [16] showed that pretreatment of mice by intracisternal administration of TNFα reduced infarct volume paradoxically without an inhibitor.

Active, soluble TNFα is derived through proteolytic cleavage from a transmembrane precursor by TNF convertase (TACE), a member of the disintegrin and metalloproteinase (ADAM) group of enzymes. Among neural cell types, soluble TNF is secreted robustly by microglia and astrocytes [17,18] but is also expressed by neurons and steroid-producing cells of the adrenal *zona reticularis*. Once released, soluble TNF circulates as a homotrimer that will bind with one of two distinct receptors of 55-kDa (TNFR1) or 75-kDa (TNFR2) molecular weight. The complex interactions of both soluble and transmembrane TNFα with the two TNF receptors have been reviewed [19]. The first receptor, p55/TNFR1, may bind either form of TNFα before dissociating from an endogenous inhibitor and recruiting the TNF receptor-associated protein death domain (TRADD). Once complexed, p55/TNFR1-TRADD may either facilitate apoptosis by activation of caspase-8 or enhance cell survival through disinhibition of the nuclear transcription factor, NFκB. The second receptor, p75/TNFR2, becomes active only upon binding transmembrane TNFα and will develop a low-affinity interaction with TRADD. The p75/TNFR2-TRADD construct is less stable than p55/TNFR1-TRADD and is considered to have a more focused role in cell survival by upregulating anti-apoptotic factors or preventing activation of caspase-8. Certain steps in the cascade, such as the activation of caspase-8 to induce apoptosis and the release of NFκB to generate pro-survival elements, arise upon stimulation of the same receptor (p55/TNFR1) and may constitute a natural check-and-balance system that determines the ultimate fate of the cell.

Our previous work suggests that the apparent duality of effect attributed to TNFα is controlled by interaction between the cytokine ligand and p55/TNFR1 or p75/TNFR2. We demonstrated that inactivation of both receptors in double-knockout mice causes exacerbation of the excitotoxic effects of kainic acid in the CA_3 _region of the hippocampus and expansion of infarct volume after middle cerebral artery occlusion (MCAO) [20]. We further highlighted the role of p55/TNFR1 as a gatekeeper molecule by showing that knock-out mice lacking only this receptor had significantly greater neuronal injury after kainic acid injection or MCAO [21].

In this paper, we describe the first application of focal cerebral ischemia in a unique transgenic rat overexpressing the murine TNFα gene. We present the results of a feasibility experiment testing the hypothesis that chronic elevation of TNFα in brain increases infarct volume after focal cerebral ischemia. We also report on comparison of TNFα-transgenic (TNFα-Tg) rats with non-transgenic (non-Tg) controls to determine if overexpression of the TNFα gene alters the vascular anatomy of the cerebral cortex, the circulatory response of the brain to ischemia, or post-ischemic cellular apoptosis. Our overall goal in performing this work was to determine how elevated levels of TNFα protein, such as may be observed in human brain after stroke, will modify cerebral ischemic injury.

## Methods

### Construction of the transgenic animal

The TNFα-Tg rat was constructed in the research facility of Xenogen Biosciences in Cranbury, New Jersey. Construction began by isolation of the murine TNFα gene from a 129 SV mouse genomic library as described previously [22]. The 2.8 kb EcoRI fragment containing the murine TNFα promoter and the entire coding region of the corresponding murine TNFα gene was ligated to the 0.77 kb EcoRI-PstI/SalI fragment containing the 3'-untranslated region and polyadenylation signal of the human β-globin gene [23]. The BamHI-SalI fragment was microinjected into fertilized eggs of Sprague-Dawley (S-D) rats using standard techniques [24]. TNFα-Tg males were bred to female, wild type S-D rats. Approximately half of the resulting offspring carried the murine TNFα gene, as confirmed by polymerase chain reaction (PCR) genotyping with primers specific for the murine TNFα gene, 5'-GGG GAA AGG TGG TAT CTC-3' (sense primer) and 5'-CGC TTT CTT GCT GTC CAA-3' (antisense primer) applied to total cellular DNA isolated from tail snip tissue.

### Measurement of TNFα mRNA and protein

The Institutional Animal Care and Use Committee of the University of Kentucky approved all experiments performed with the TNFα-Tg rat. To analyze TNFα mRNA levels in TNFα-Tg and non-Tg brain, reverse transcriptase-PCR (RT-PCR; Ambion, Austin, TX) was performed on total RNA isolated from one cerebral hemisphere. An enzyme-linked immunosorbent assay (ELISA) was used to detect aggregate mouse and rat TNFα in the remaining cerebral hemisphere (R & D Systems, Minneapolis, MN).

To determine the contribution of the native rat gene to the mRNA response, we screened for rat TNFα mRNA in different brain regions, external organs, and serum in a second set of animals. Samples were homogenized and total RNA was extracted with an RNeasy Mini Kit (QIAGEN Inc., Valencia, CA). The Quantikine rat TNFα probes and Calibrator Kit (#RN510) was then used with an mRNA base kit (#RN000) of R & D Systems to quantify rat TNFα mRNA. Parallel samples were homogenized in phosphate-buffered saline (PBS), centrifuged, and total rat and mouse TNFα was measured by ELISA (R & D Systems; Quantikine M catalog #MTA00).

To measure TNFα protein and mouse or rat mRNA in small quantities of brain tissue (= 50 mg) obtained from ischemic animals, we developed a micro-sample preparation technique and refined our protein ELISA and mRNA quantification methods. Tissue samples obtained from the border zone approximating the ischemic penumbra and the infarct core in the rat brain were homogenized in Mammalian Tissue Lysis Buffer (#C3228; Sigma Chemical Company, St. Louis, MO) consisting of a dialyzable mild detergent (bicine), 150 mM NaCl, and a cocktail of protease inhibitors (Roche). The homogenates were centrifuged at 12,000 × g for 10 minutes at 4°C. Protein concentration was quantified with the BCA protein assay kit (#23225, Pierce Chemicals, Rockford, IL).

For measurement of TNFα protein in small tissue samples, we used an ELISA kit (eBioscience, San Diego, CA) containing a monoclonal antibody that can detect both mouse and rat TNFα protein in the range of 10 pg/ml – 10 ng/ml. To measure rat or mouse mRNA in small tissue samples, we extracted total RNA with the RNeasy Mini Kit and used the same Quantikine mRNA base kit described above. Quantikine rat TNFα mRNA probes and Calibrator Kit (#RN510) and Quantikine mouse TNFα mRNA probes and Calibrator Kit (#RN410) with Quantikine mRNA base kit (#RN000) of R & D Systems were used to quantify mouse and rat TNFα mRNA. After completion of this assay, mouse or rat TNFα mRNA was quantified in attomoles/ml/1 μg total RNA and expressed as a ratio (mouse/rat TNFα mRNA).

### Western blot analysis for TRADD protein

We measured expression of TRADD protein, derived upon interaction of active, soluble TNFα with p55/TNFR1, to demonstrate that TNFα protein retained biological activity in the ischemic brain of the TNFα-Tg rat. Micro-samples of brain tissue were homogenized and prepared as described for measurement of TNFα protein. The expression of TRADD protein was determined by western blot analysis using a monoclonal anti-TRADD antibody (BD Biosciences, USA). Aliquots of brain homogenate (30 μg of protein) were loaded onto 10% SDS-PAGE gels for electrotransfer onto nitrocellulose membranes. After transfer, the membranes were blocked by incubation in 5% BSA in TRIS-buffered saline with 0.01% Tween-20 for one hour at room temperature and were washed three times (10 minutes each) with wash buffer (1 × TBS, 0.05% Tween-20). The membranes were then incubated with primary antibody (anti-TRADD monoclonal antibody; #610572, BD Biosciences Pharmingen, San Diego, CA) at a dilution of 1:250 for one hour at room temperature. The nitrocellulose membrane was then washed and incubated with horseradish peroxidase-conjugated goat anti-mouse IgG (1:1,000; #34080, Cell Signaling Technology, Danvers, MA) for another hour at room temperature. After three additional washes in TBS/Tween-20, immunoreactive bands were developed using an ECL chemiluminescence substrate (#34080, Pierce). A densitometry image analysis system (Kodak 2000RT) was used for quantification of band intensities on X-ray film. To screen for gel loading errors, the western blots were stripped and reprobed with an antibody to β-actin, a constitutively expressed protein.

### Immunohistochemical studies

For immunohistochemical studies confirming cell identity and intracellular expression of TNFα, we prepared 30-μm coronal brain slices and applied methods described in our previous publications [25-27]. We used a rabbit polyclonal antibody to identify both rat and mouse TNFα (AMC3012, Biosource International, Camarillo, CA; 1:2000 dilution). We chose NeuN (MAB377, Chemicon International, Temecula, CA; mouse monoclonal; 1:1000 dilution) to immunolabel neuronal nuclear protein, anti-GFAP (anti-glial fibrillary acidic protein; MAB3402, Chemicon; mouse monoclonal; 1:1000 dilution) to identify astroglia, CD11b/OX-42 (MAB1405, Chemicon; mouse monoclonal; 1:1000 dilution) to label microglia and macrophages, and anti-APC/Ab-7 (anti-oligodendroglial cell body; OP80, Oncogene Research Products; mouse monoclonal; 1:1000 dilution) to identify oligodendrocytes. To identify what type of neural cell was expressing TNFα in the transgenic animal, co-localization studies were performed by incubating coronal brain sections with the anti-TNFα antibody and NeuN, anti-GFAP, CD11b/OX-42, or anti-APC/Ab-7 labeled with fluorophores.

### Suture-occlusion model of focal cerebral ischemia

Because the murine TNFα gene was expressed within S-D rats, we chose the Zea Longa technique [28] for suture-occlusion of the MCA already validated in this animal strain. Male transgenic and non-Tg rats of 250–350 gm body weight were subjected to one hour of suture-occlusion by our modification [29] of the original Zea Longa method. Each animal was fasted overnight in preparation for surgery and then anesthetized by IP injection of chloral hydrate (350 mg/kg) and xylazine (4 mg/kg). Rectal and temporalis muscle temperatures were maintained at 36.5–37.5°C by external warming. A suture-occluder prepared by the method of Belayev and others [30] was advanced retrograde through the external carotid artery and into the internal carotid artery to occlude the MCA. After one hour, the suture-occluder was withdrawn. In each rat, the left femoral artery was reversibly cannulated for assessment of mean arterial blood pressure (MABP) and blood gases before, during, and after suture-occlusion.

### Measurement of infarct volume

Infarct volume was measured 24 hours or 7 days after removal of the suture-occluder from the MCA in ischemic animals. Rats prepared for measurement of infarct volume were re-anesthetized by IP injection of the same combined doses of chloral hydrate and xylazine used as anesthesia for surgery to achieve focal cerebral ischemia. For brain sampled after 24 hours of post-ischemic reperfusion, each anesthetized animal was euthanatized for staining of coronal brain sections with triphenyltetrazolium chloride (TTC) [31]. Total percent infarct volume was quantified using NIH Image v. 1.62 on a computer-assisted imaging system described in our previous work [26,29]. Infarct volume was corrected for ischemic edema by the method of Swanson and co-authors [32].

For measurement of infarct volume after 7 days of post-ischemic reperfusion, animals were re-anesthetized by IP injection of chloral hydrate/xylazine and perfused transcardially with 30 ml of cold, heparinized 0.1 M PBS (pH 7.4), followed by 60 ml of 4% paraformaldehyde in PBS. Each brain was removed *en bloc *and placed in 4% paraformaldehyde/0.1 M PBS at 4°C for 24 hours. The fixed brain specimen was then washed externally with PBS × 3 (10 minutes each) at 4°C. The brain specimen was blocked and immersed in 20% sucrose/0.1 M PBS at 4°C for 24 hours. The fixed, sucrose-protected brain was mounted on a metal chuck with cryostat embedding medium. The mounted brain was frozen in powdered dry ice and brought to ambient temperature within the cryostat (-21°C) before 80-μm coronal sections were cut to encompass the anticipated infarct volume. Every fifth section was mounted directly onto glass microscopic slides that were dried on a slide warmer at 40°C. The dried sections were defatted in xylene overnight before rehydration through gradient alcohol solutions to water. The resulting sections were then stained in 5% cresyl violet acetate for 2 minutes, rinsed in water, dehydrated through gradient alcohol solutions to xylene, and coverslipped. Total percent infarct volume was quantified using NIH Image v. 1.62 on the same computer-assisted imaging system used for quantifying 24-hour infarct volume, but without correction for tissue edema.

### Assessment of neural cell death after focal cerebral ischemia

We employed both qualitative and quantitative methods to evaluate apoptotic cell death in the brains of TNFα-transgenic and non-Tg rats after ischemic injury. For qualitative determination of what cell type was undergoing caspase-3 activation after ischemia, we adapted the method of McEwen and Springer [33] from its original application in experimental spinal cord trauma to our model of suture-occlusion of the MCA. Twenty-four hours after reversal of ischemia by withdrawal of the suture-occluder, each rat was re-anesthetized by IP injection of chloral hydrate/xylazine and perfused transcardially with 20 ml of cold, heparinized 0.1 M PBS (pH 7.4), followed by 40 ml of 4% paraformaldehyde in PBS. The brain was removed *en bloc *and placed in 4% paraformaldehyde in PBS at 4°C for one hour. The fixed brain specimen was then washed externally with PBS × 3 (10 minutes each) at 4°C. The brain specimen was then blocked and immersed in 20% sucrose-PBS at 4°C for 48 hours, before being frozen rapidly on dry ice and embedded in tissue freezing medium (Triangle Biomedical Sciences, Durham, NC). The frozen brain was then cut into 20-μm coronal sections on a cryostat (Microm Laborgerate, Walldorf, Germany). Consecutive sections taken from both TNFα-Tg and non-Tg rats were mounted in pairs on a series of charged glass slides (Superfrost Plus; Fisher Scientific, Pittsburgh, PA).

The sections were air-dried and stored at -20°C before preparation for fluorescence immunohistochemistry, all procedures for which were performed at room temperature except as noted. All sections were air-dried for 30 minutes after removal from cold storage, washed repeatedly in PBS for one hour, and pre-incubated for 30 minutes in a blocking solution comprised of 5% normal goat serum (NGS) and 0.05% Triton X-100 in PBS. The blocking solution was removed and sections were incubated in a humidified chamber overnight at 4°C in PBS that contained 5% NGS and one of the following sets of primary antibodies: (a) rabbit anti-human/mouse activated caspase-3 IgG (polyclonal, 1:1,000; R & D Systems, Minneapolis, MN), (b) mouse anti-neuronal nuclei (NeuN) IgG (monoclonal, 1:600; Chemicon International, Temecula, CA) to identify neurons, or (c) anti-glial fibrillary acidic protein (GFAP; monoclonal, 1:600; Sigma, St. Louis, MO) to localize astrocytes. To test for nonspecific staining by the secondary antibodies, additional slides were processed in a similar fashion with the primary antibodies excluded. All slides were then washed repeatedly in PBS for one hour before being incubated in the dark for a second hour in PBS that contained 5% NGS and the following fluorescent secondary antibodies: Cy-3-conjugated goat anti-rabbit IgG (polyclonal, 1:1,600; Jackson ImmunoResearch Laboratories, West Grove, PA) and AlexaFluor 488 goat anti-mouse IgG (monoclonal, 1:600; Molecular Probes, Eugene, OR). The secondary antibody solution was removed and the slides were washed repeatedly in PBS for 10 minutes. Tissue sections were immediately coverslipped with ProLong Anti-Fade (Molecular Probes) and stored in the dark at 4°C to retard fading of the fluorescent labels. A Zeiss AxioPlan microscope (Zeiss; Oberkochen, Germany) was used to review the tissue sections after processing. Co-localization of immunoreactivity was performed with Photoshop (Adobe Systems; San Jose, CA) to superimpose paired images (40×) showing the fluorescent secondary antibodies identifying anti-active caspase-3 and the cell type of interest.

To determine if neural cell death was preceded by caspase-3 activation, we quantified the activity of this pro-apoptotic enzyme in ischemic brain. TNFα-Tg and non-Tg rats underwent MCAO by suture-occlusion for one hour. After 3 or 24 hours of post-ischemic reperfusion, each animal was re-anesthetized by IP injection of chloral hydrate and was perfused transcardially with 30 ml of 15% India ink (v/v) diluted in normal saline, as per our published method [27]. The brain was removed *en bloc*, chilled, and cut into 2-mm sections. Normally perfused brain was darkened by the ink, whereas unreperfused tissue remained pale. The infarct core within each slice was dissected and snap-frozen, as was a thin rim of partially stained tissue at the interface between normal and unreperfused cortex that approximated the ischemic penumbra. The resulting tissue samples were prepared by the micro-sample method described above and caspase-3 activity was measured with a caspase-3 substrate AC-DEVD-AFC Fluorometric Assay Kit (R & D Systems). All procedures were carried out according to the manufacturer's instructions. Equal amounts of protein samples were mixed with caspase-3 reaction buffer containing the fluorescent peptide AC-DEVD-AFC, pipetted in duplicate in a 96-well microtiter plate, and incubated at 37°C for 180 minutes. Caspase-3 mediated cleavage of AC-DECD-AFC into free AFC was measured using a fluorescent micro plate reader (LS50B, Perkin Elmer) at a wavelength excitation of 400 nm and emission of 505 nm. Readings were obtained serially at 0, 30, 60, 120, and 180 minutes. The change in fluorescence (Δ fluorescence units) was calculated by subtracting the minimal (t = 0 minutes) from the maximal (t = 180 minutes) reading and the caspase-3 activity in each sample was calculated in Δ fluorescence units/minute/100 mg protein.

To confirm that intracellular caspase-3 activation preceded apoptotic cell death, we performed a quantitative assay for DNA fragmentation after focal cerebral ischemia. For this technique, we adapted the method of Shimizu and colleagues for measurement of DNA fragmentation in ischemic gerbil hippocampus [34]. TNFα-Tg and non-Tg rats underwent MCAO by suture-occlusion for one hour. After 3 or 24 hours of post-ischemic reperfusion, each animal was re-anesthetized by IP injection of chloral hydrate and was perfused transcardially with India ink. The brain was removed *en bloc*, chilled, cut into 2-mm sections, and sampled as described in the preceding paragraph. Frozen tissue samples were homogenized with a glass tissue grinder in isolation buffer comprised of 215 mM mannitol, 75 mM sucrose, 0.1% BSA, 1 mM EGTA (pH 7.2), and 20 mM HEPES (pH:7.2). Each homogenate was then centrifuged at 3,000 × g for 5 min at 4°C to remove whole DNA within intact cellular nuclei. The resulting supernatant was recentrifuged at 13,000 × g for 10 min at 4°C to discard a crude mitochondrial pellet. The second supernatant enriched for cytosolic, fragmented DNA was aliquotted and stored at -80°C. Protein concentration in the second supernatant was measured with the BCA protein assay kit (Pierce).

DNA fragmentation in the cytoplasmic supernatant was quantitatively assayed by antibody-mediated capture and detection of histone-associated DNA fragments (mono- or oligonucleosomes; Cell Death Detection ELISA Plus kit; Roche Molecular Biochemicals, Mannheim, Germany). Briefly, a 20-μl aliquot of the cytoplasmic supernatant was used in the ELISA following the manufacturer's standard protocol. Optical density values of absorbance at 405 and 490 nm (reference wavelength) were determined with a microplate reader (SpectraMAX 340PC, Molecular Device) after incubation with a peroxidase substrate. Background values (incubation buffer alone) were subtracted from each sample. All samples were run in duplicate. The direct reading units of OD values were converted to mU (absorbance = 1000 mU). The results were shown as mU (arbitrary unit) per mg protein.

### Measurement of regional cerebral perfusion and quantification of vascular density

We used laser-Doppler flowmetry (LDF) to measure regional cerebral perfusion in TNFα-Tg rats or non-Tg controls during focal cerebral ischemia and post-ischemic reperfusion. Following our previously reported method [29], each animal underwent craniotomy for placement of a 2-mm laser-Doppler probe over intact dura approximately 3 – 5 mm posterior to bregma and 4 mm lateral to midline. The probe was positioned over a cortical region affected by MCAO, from 4.2 mm anterior to 5.8 mm posterior in relation to bregma, avoiding large pial vessels to obtain reproducible, stable readings [35]. Baseline measurements were taken every five minutes during 10 minutes of pre-ischemic baseline, 60 minutes of MCAO, and the first 30 minutes of post-ischemic reperfusion. Results were expressed as percent of pre-ischemic baseline.

For quantification of vascular density, we employed a stereological method used previously to study vascular changes in rodent hippocampal dentate gyrus [36]. Male TNFα-Tg and non-Tg rats (n = 3 per group) that were not subjected to MCAO were anesthetized by IP injection of sodium pentobarbital (55 mg/kg) and underwent thoracotomy to gain access to the heart. After cross-clamping of the descending aorta, each animal was infused with 20 ml undiluted Higgins black waterproof India ink #4115 (Sanford, Bellwood, IL). The infusate was delivered through a 20-gauge needle inserted directly into the beating left ventricle, using hand-applied pressure. The infused animal was decapitated; the brain was then removed *en bloc *and post-fixed in 4% paraformaldehyde for 48 hours. It was then cryoprotected in 20% sucrose (w/v) for three days under constant, mild agitation. Coronal sections of 50-μm thickness were cut with a freezing microtome throughout the rostral-caudal extent of a specified region of the cortex extending from the septal area (inter-aural level 10. 7) to the occipital region (inter-aural level -0.3) [37]. The Bioquant Image Analysis System (Nashville, TN) was used to analyze 12 equidistant sections taken from every animal. For each section, an unbiased method [38] was employed to select a region of interest (ROI; 5.26 mm^2^) within the cortex. With the Bioquant device, a thresholding routine was used to estimate the percent area of the ROI occupied by ink-labeled blood vessels. The vascular percent areas within the ROIs in the 12 coronal sections were averaged to derive a mean value for each animal.

### Statistical analysis

All physiological measurements, levels of TNFα mRNA and protein in brain, and infarct volumes by experimental group were expressed as means ± SD. Unpaired *t *tests were used for comparisons of group means for infarct volume, cortical vascular density, and selected physiological measurements (pre-ischemic whole blood glucose and hematocrit). All other physiological data, including measurements of cortical perfusion by LDF, were obtained serially in each rat and were compared by two-way (experimental group and sampling time) analysis of variance (ANOVA) with repeated measures. For comparison of TNFα mRNA or protein in different brain regions, external organs, and serum in TNFα-Tg and non-Tg animals, ANOVA was used with Fisher's protected least significant difference post hoc test that corrects for multiple comparisons. TRADD protein levels and mouse/rat TNFα mRNA ratios were also compared with ANOVA/Fisher's test. Statistical significance was considered at p ≤ 0.05.

## Results

### Production and characterization of the TNFα-Tg rat

**Figure 1:**
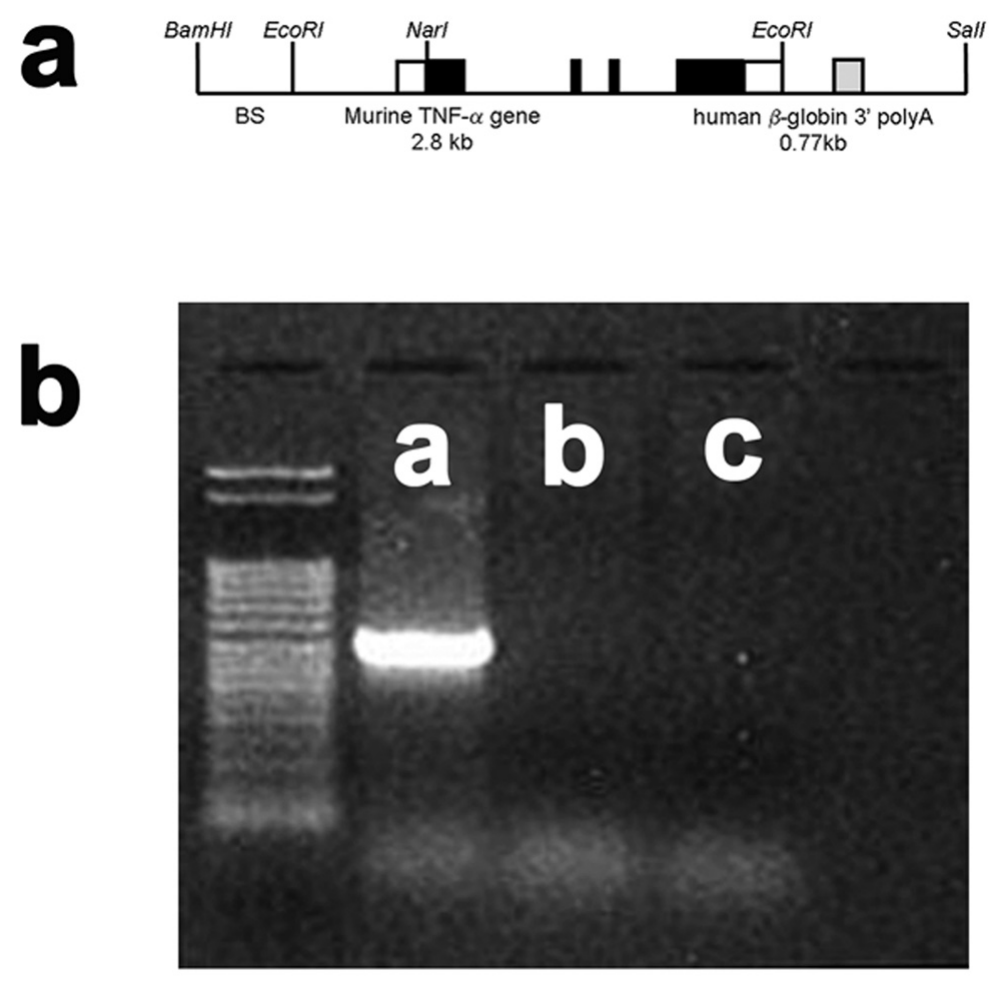
**Structure of the murine TNFα transgenic construct and confirmation of genotype**. The structure of the murine TNFα transgenic construct is illustrated in Panel a. An example of polymerase chain reaction (PCR) products stained with ethidium bromide to confirm the genotype of the TNFα-transgenic (TNFα-Tg) rat is shown in Panel b. Clear boxes in the drawing of the transgenic construct represent untranslated sequences from the mouse TNFα gene, black boxes indicate the coding region of the TNFα gene, and the gray box (including the remainder of the *EcoRI *to *SalI *fragment) represent the human β-globin 3' fragment. BS indicates pBluescript vector. Panel b shows PCR products in tail snip tissue taken from a TNFα-Tg rat (Lane a), a non-transgenic littermate (Lane b), and a Sprague-Dawley rat (Lane c). A 501 bp fragment of the murine TNFα gene was detected only in the transgenic rat.

The murine TNFα promoter and the entire coding region of the corresponding murine TNFα gene (Figure 1, Panel a) were introduced into the TNFα-Tg rat. Weanling pups underwent tail snip on post-natal Day 21 to obtain tissue for isolation of total cellular DNA. The 501-kb fragment of the murine TNFα gene was present only in the transgenic animal, allowing confirmation of genotype by PCR (Figure 1, Panel b).

**Figure 2:**
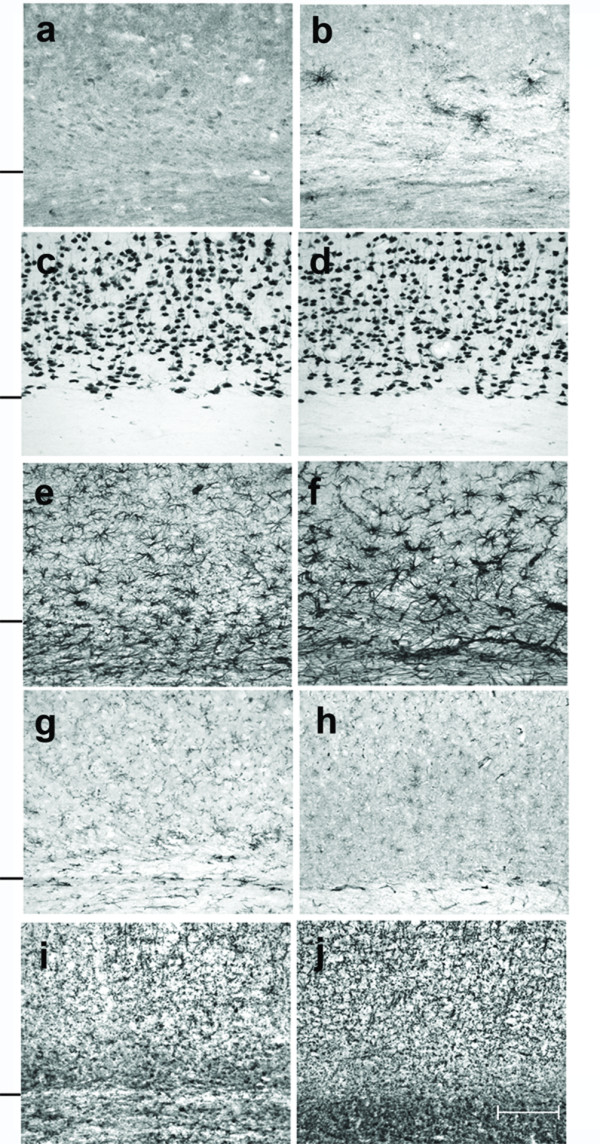
**Cellular expression of TNFα protein in transgenic and non-transgenic rat brain**. This photographic collage shows cellular expression of TNFα protein and compares the brain anatomy of transgenic and non-transgenic (non-Tg) rats that were not subjected to ischemic injury. Coronal brain sections were incubated with primary antibodies identifying mouse or rat TNFα protein (Panels a and b), neurons (Panels c and d), astroglia (Panels e and f), microglia/macrophages (Panels g and h), or oligodendroglia (Panels i and j). All light microscopic images were taken at 20× magnification. Transgenic rat brain is shown in Panels b, d, f, h, and j. All remaining images are of brain in non-Tg littermates. Cells that were immunoreactive for TNFα appeared only in transgenic animals, as shown in Panel b. A hash mark drawn to the left of each pair of 20× images represents the interface between neocortex and internal capsule. Scale bar = 100 μm

**Figure 3:**
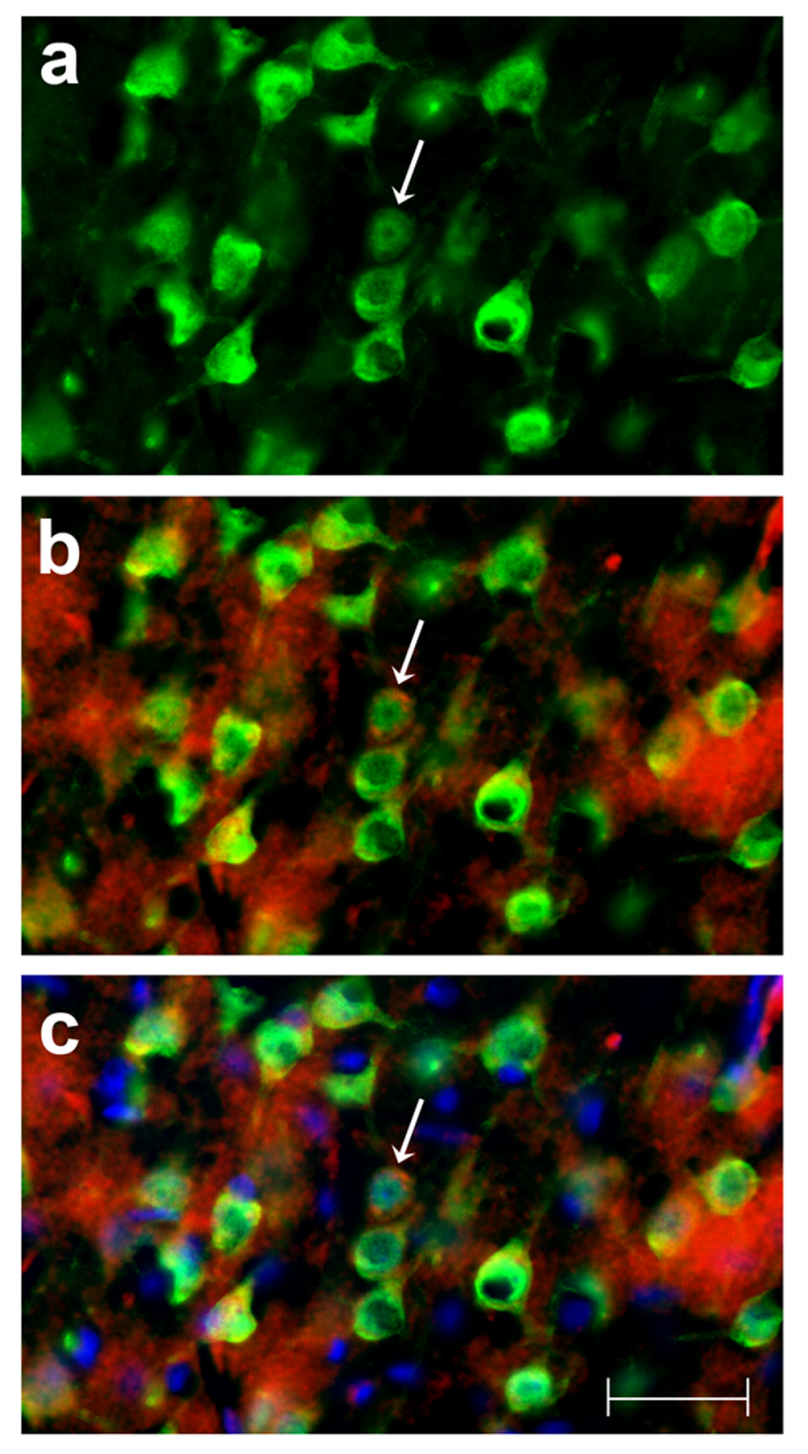
**Identification of neural cell type containing TNFα protein**. This photographic collage shows the result of co-localization studies identifying the neural cell type containing TNFα protein in the brain of the transgenic animal. All images were taken of TNFα-transgenic rat brain that had not been subjected to ischemic injury. Coronal brain sections were incubated with primary antibodies identifying mouse or rat TNFα protein and neurons. All images were made by fluorescence microscopy at 40× magnification. In Panel a, cortical neurons in TNFα-Tg rat brain are immuno-labeled with the anti-neuronal antibody NeuN tagged with a green fluorophore. In Panel b, co-localization images are merged to show that several NeuN-labeled neurons (green) express TNFα protein immuno-labeled with anti-mouse/rat TNFα antibody tagged with a red fluorophore. In Panel c, co-localization images are merged to show NeuN-labeled neurons (green), TNFα protein (red), and cellular nuclei labeled with Hoechst stain (blue). A representative neuron with cytoplasmic inclusions labeled to show expression of TNFα protein is identified in each Panel (arrow). In Panel c, nuclei of several non-neuronal cells that do not express TNFα protein are shown. Scale bar = 50 μm

Cells that were immunoreactive for TNFα were found only in the brain of the TNFα-Tg rat. By histological examination, the neural anatomy of the TNFα-Tg rat appeared similar to that of non-Tg littermates (Figure 2, Panels a – j) when not subjected to ischemic injury. Co-localization studies (Figure 3, Panels a – c) showed overlap of signal between mouse TNFα protein and a neuronal marker, NeuN. This result identifying the rat neuron as the primary neural cell expressing TNFα protein was confirmed in three TNFα-Tg rats from three different litters. Every fifth coronal section was sampled in each of the three transgenic animal brains.

**Figure 4:**
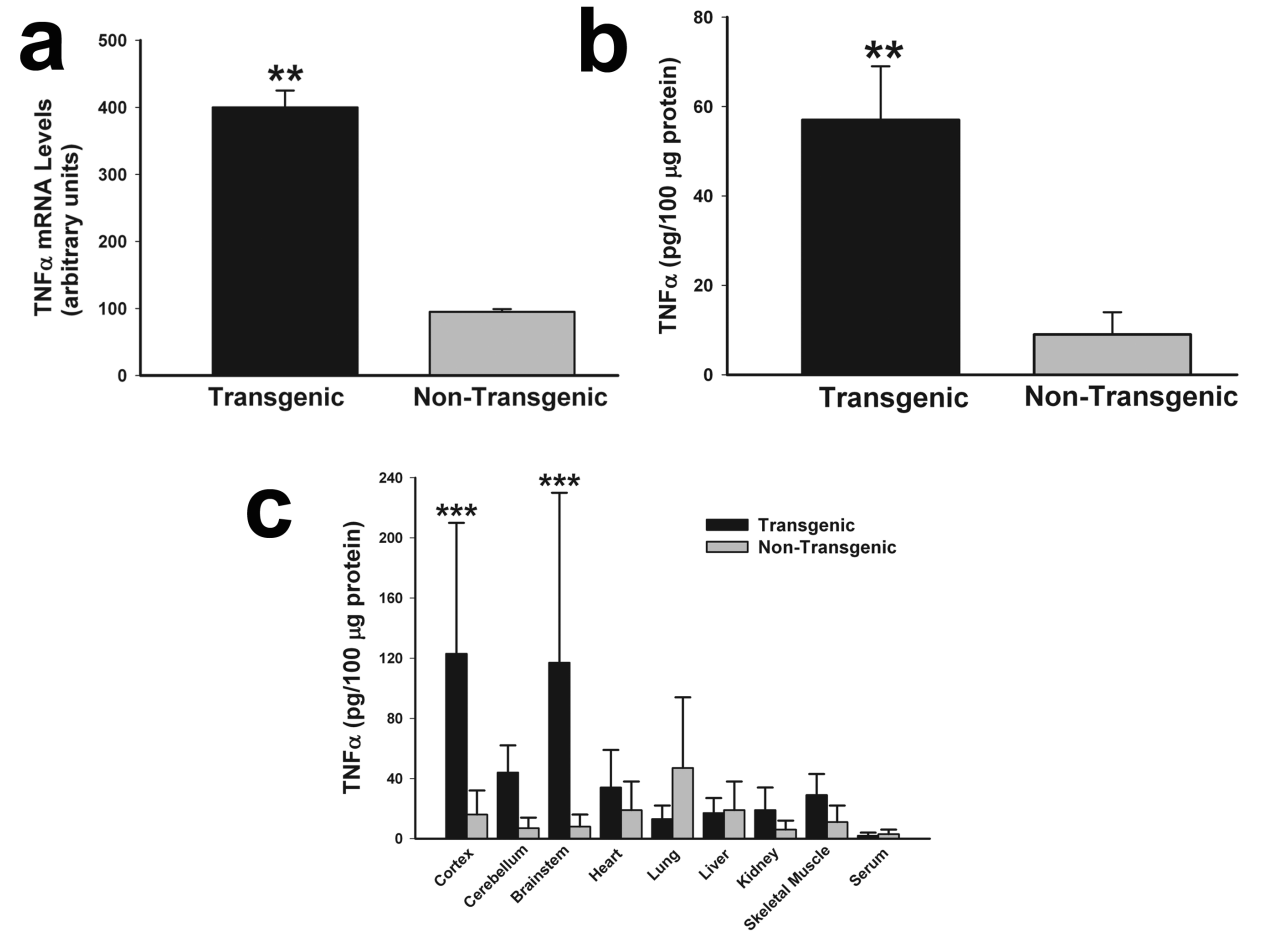
**Organ-specific expression of TNFα mRNA and protein in transgenic and non-transgenic rats**. Two bar graphs show aggregate rat and mouse TNFα mRNA (Panel a) or protein (Panel b) in brain homogenates prepared from transgenic animals and non-transgenic (non-Tg) littermates (n = 3 in each group). A third bar graph (Panel c) shows TNFα protein levels in three brain compartments, various external organs, and randomly obtained serum samples from a second set of transgenic and non-Tg animals (n = 3 in each group). No animal was subjected to ischemic brain injury. In Panel a, the mean ± SD level of TNFα mRNA was significantly higher in transgenic rats (p ≤ 0.01; unpaired *t *test). The mean TNFα protein level was also significantly elevated in transgenic animals, as shown in Panel b (p ≤ 0.01; unpaired *t *test). When TNFα protein was surveyed in multiple organs and in serum, its content in cortex and brainstem was at least fivefold higher than in non-Tg animals (p ≤ 0.001; ANOVA/Fisher's test). **p ≤ 0.01; ***p ≤ 0.001

Murine TNFα mRNA and protein were overepressed abundantly in the TNFα-Tg rat. In Figure 4, multiple panels show the results of assays for TNFα mRNA and protein in transgenic animals and non-Tg littermates that were not subjected to ischemic injury. The aggregate level of rat and mouse TNFα mRNA assayed by RT-PCR (Panel a) was fourfold higher in TNFα-Tg than in non-Tg rats (n = 3 per animal type; p ≤ 0.01; unpaired *t *test). The mean level of rat TNFα mRNA in the cortex of the transgenic rat, 1.3 ± 0.5 attomoles/μg total RNA, was not significantly different from 0.8 ± 0.1 in the non-Tg animal and was no more remarkable in cerebellum and brainstem (data not shown). The aggregate level of rat and mouse TNFα protein in whole brain homogenate (Panel b) was fivefold higher in TNFα-Tg rats (n = 3 per animal type; p ≤ 0.01; unpaired *t *test). In Panel c, we show the results of a second ELISA assaying for rat and mouse TNFα protein in three brain regions (cortex, cerebellum, and brainstem), a variety of organs outside the brain, and in randomly sampled serum in both TNFα-Tg and non-Tg animals (n = 3 per animal type). Cortex and brainstem in the TNFα-Tg rat had significantly higher levels (p ≤ 0.001; ANOVA/Fisher's test). The level of aggregate TNFα protein in the cerebellum of the TNFα-Tg animal (44 ± 18 pg/100 μg protein) was higher than in the non-Tg rat (7 ± 3) but did not reach statistical significance. It is noteworthy that the levels of aggregate TNFα were almost two-fold higher in the heart, kidney, and skeletal muscle of the transgenic animal in comparison to non-Tg rats, although these differences failed to reach statistical significance. We attributed the difference in quantity of aggregate TNFα protein shown in Panels b and c to the dilutional signal-to-noise loss in the brain homogenate.

### Focal cerebral ischemia in the TNFα-Tg rat

**Figure 5:**
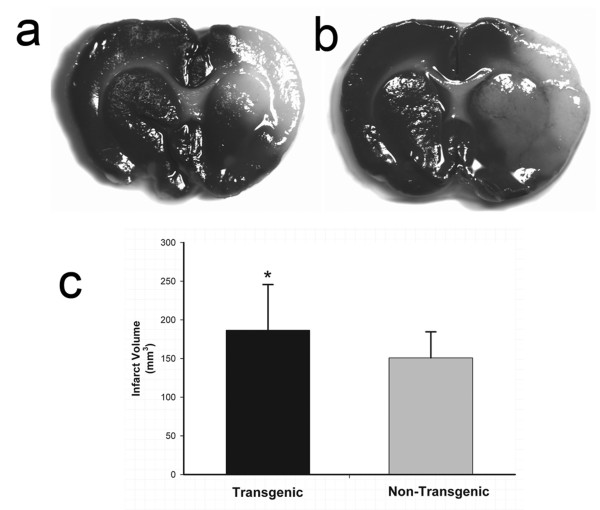
**Quantification of brain infarct volume in TNFα-transgenic and non-transgenic rats at 24 hours**. Representative infarcts are shown in the brain of a non-transgenic (Panel a) and a TNFα-transgenic (TNFα-Tg) rat (Panel b). In Panel c, a bar graph shows mean percent infarct volume in both TNFα-Tg and non-Tg rats. All animals underwent suture-occlusion of the right middle cerebral artery for one hour followed by 24 hours of post-ischemic reperfusion. The 1-mm coronal brain sections in Panels a and b were stained for triphenyltetrazolium chloride. Panel a shows infarction (blanched tissue) confined to the right cortical mantle and a portion of the underlying striatum in a non-Tg rat. In the transgenic animal shown in Panel b, there is blanching of tissue in the right cortical mantle that indicates completed infarction and mottled discoloration throughout the entire ipsilateral striatum that is a maturing infarct. Panel c shows that mean ± SD infarct volume in the transgenic group (n = 12) was significantly greater than that of the non-Tg group (n = 17; *p ≤ 0.05; unpaired *t *test). All volume measurements were corrected for tissue edema.

We induced focal cerebral ischemia by unilateral MCA suture-occlusion in TNFα-Tg rats and non-Tg littermates. Infarct volumes were calculated after 24 hours or 7 days of post-ischemic reperfusion. The 24-hour infarct volumes were derived by computer-assisted planimetry of digitized, TTC-stained coronal brain slices; all data sets were corrected for edematous tissue expansion. The 7-day infarct volumes were obtained by the same approach but were derived from examination of coronal brain sections stained with cresyl violet to identify Nissl substance. After 24 hours, TNFα-Tg rats had larger infarctions demonstrated by TTC staining of coronal brain slices (Figure 5, Panels a and b). In the TNFα-Tg rat (n = 12), mean infarct volume ± SD was larger than in non-Tg littermate controls (n = 17; p ≤ 0.05; unpaired *t *test; see Figure 5, Panel c). Table 1 shows all physiological data obtained on randomly sampled animals subjected to MCAO and measurement of infarct volume by TTC staining. There was no significant group-by-time interaction on two-way ANOVA of any variable. These results support our conclusion that between-group differences in infarct volume could not be attributed to unique susceptibility of the TNFα-Tg rat to hypoxemia or hypercarbia during or after ischemia.

**Table 1:** Physiological Data.

	**Measurements**						
**Test Group**	**Glucose** **(mmol/L)**	**Hematocrit** **(%)**	**Core Temperature** **(°C)** **(temporalis muscle)**	**Mean Arterial** **Blood Pressure** **(mm Hg)**	**Arterial Oxygen** **Pressure** **(mm Hg)**	**Arterial Carbon** **Dioxide Pressure** **(mmHg)**	**Arterial** **Blood pH** **(Units)**
TNFα-Transgenic Rats (n = 7)							

Pre-ischemic Sample	4.29 ± 0.38	47 ± 1	36 ± 0.4/36 ± 0.4	99 ± 10	73 ± 6	47 ± 4	7.30 ± 0.02
Ischemic Sample	-	-	37 ± 0.2/37 ± 0.3	84 ± 13	71 ± 5	44 ± 4	7.30 ± 0.02
Post-ischemic Sample	-	-	37 ± 0.3/37 ± 0.5	84 ± 9	70 ± 3	43 ± 4	7.31 ± 0.02

Non-Transgenic Controls (n = 12)							

Pre-ischemic Sample	4.18 ± 0.80	47 ± 2	36 ± 0.6/36 ± 0.5	96 ± 13	71 ± 8	50 ± 3	7.29 ± 0.02
Ischemic Sample	-	-	37 ± 0.3/36 ± 0.3	92 ± 12	69 ± 8	48 ± 3	7.29 ± 0.02
Post-ischemic Sample	-	-	37 ± 0.4/37 ± 0.4	88 ± 14	71 ± 7	45 ± 3	7.30 ± 0.02

Arterial blood samples were obtained 15 minutes before insertion of the suture (pre-ischemic sample), immediately before its removal (ischemic sample), and after 15 minutes of reperfusion (post-ischemic sample).

**Figure 6:**
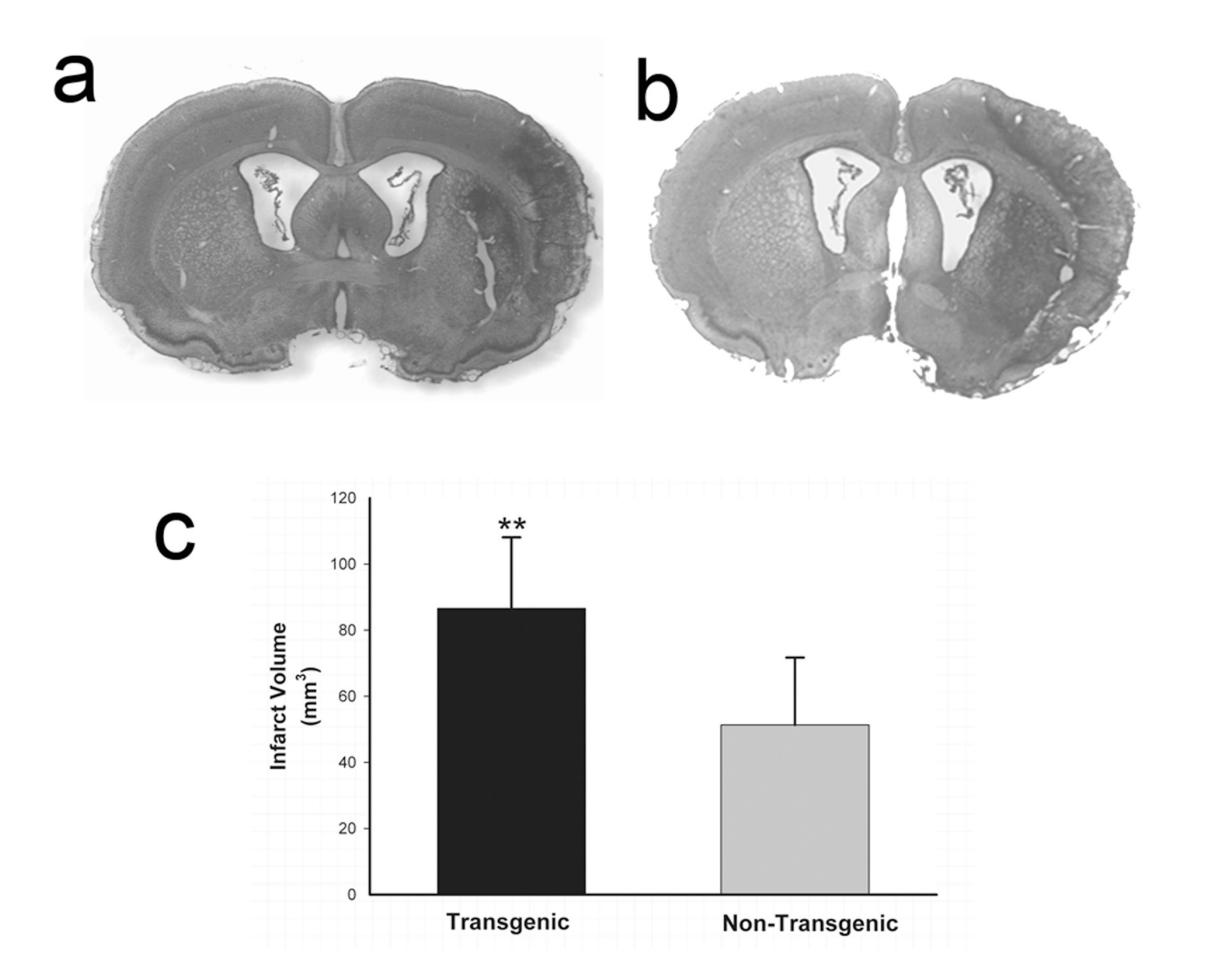
**Quantification of brain infarct volume in TNFα-transgenic and non-transgenic rats at 7 days**. Representative infarcts are shown in the brain of a non-transgenic (non-Tg; Panel a) and a TNFα-transgenic (TNFα-Tg) rat (Panel b). In Panel c, a bar graph shows mean ± SD infarct volume in both TNFα-Tg and non-Tg rats. All animals underwent suture-occlusion of the right middle cerebral artery for one hour followed by 7 days of post-ischemic reperfusion. The 80-μm coronal sections of brain shown in Panels a and b were stained with cresyl violet for Nissl substance. Panel a shows well-demarcated infarction confined to the right cortical mantle and a portion of the underlying striatum in the non-Tg rat. In the transgenic animal shown in Panel b, the infarct occupies a larger proportion of the right cortical mantle and almost all of the ipsilateral striatum. Panel c shows that mean infarct volume in the transgenic group (n = 10) was significantly greater than that of the non-Tg group (n = 11; **p ≤ 0.01; unpaired *t *test).

Recognizing that TNFα and other inflammatory cytokines rise to peak levels in rat brain within 24 – 48 hours after ischemic injury [39], we re-assessed infarct volume 7 days after reversal of MCAO to observe for a delayed effect on injury progression. We also wished to know if the difference between infarct volumes in TNFα-Tg and non-Tg rats observed at 24 hours proved resilient at 7 days. We found that TNFα-Tg rats had larger infarcts that persisted after 7 days of post-ischemic reperfusion (Figure 6, Panels a and b). Mean percent infarct volume remained higher in TNFα-Tg rats (n = 10) than in non-Tg littermates (n = 11; p ≤ 0.01; unpaired *t *test; see Figure 6, Panel c). We performed between-group comparisons of mean pre-ischemic body weight, anticipating that TNFα-Tg rats may be affected by somatic wasting that could contribute to the observed difference in infarct volume at 7 days. The pre-ischemic mean weight in TNFα-Tg rats prepared for MCAO and measurement of infarct volume at 7 days was 287.2 ± 33.5 (SD) g, compared to 310.1 ± 21 in non-Tg littermates (p = NS).

**Figure 7:**
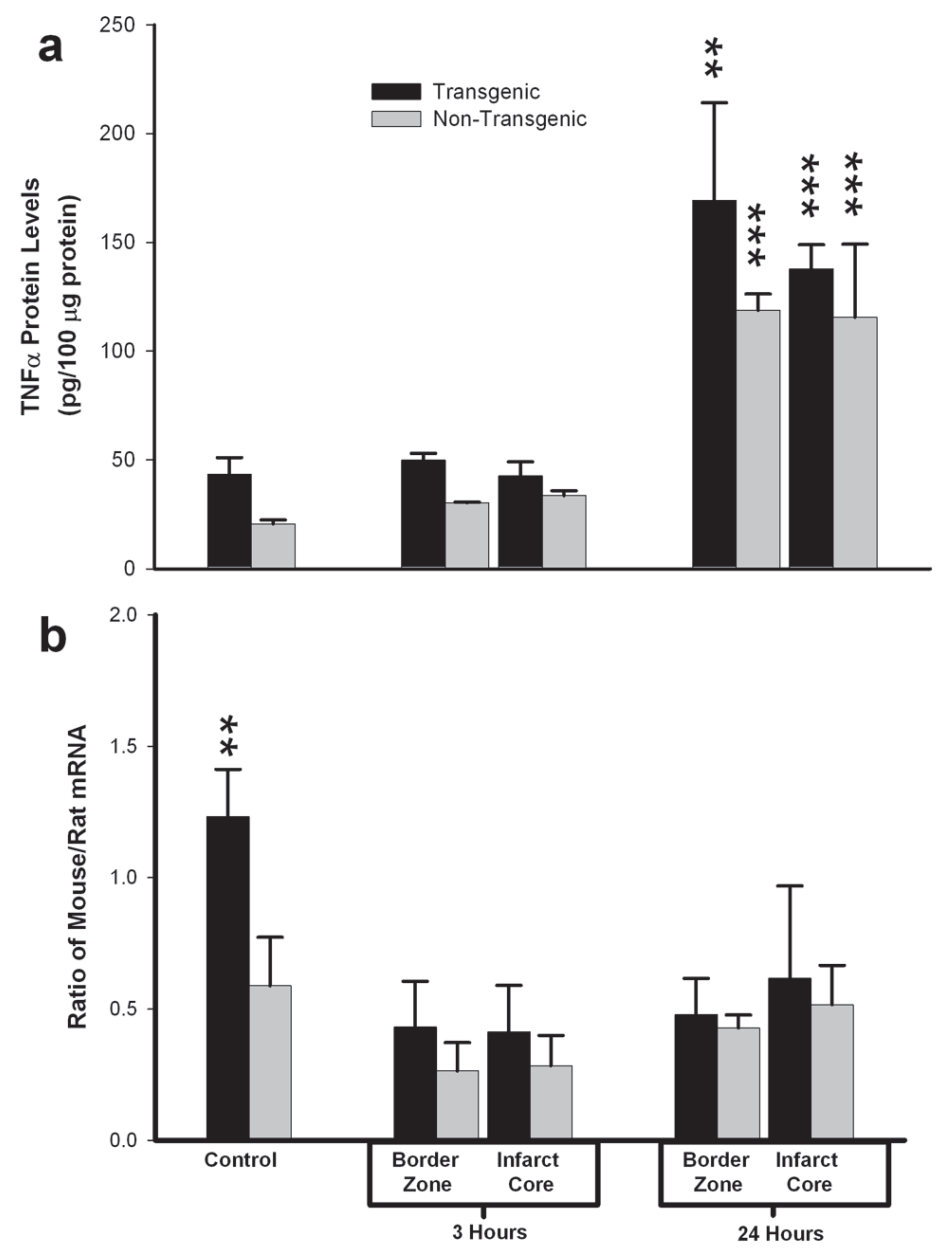
**Regional expression of TNFα mRNA and protein in brain after focal ischemia**. Two bar graphs show mean ± SD levels of TNFα protein (Panel a) and the corresponding mean ratios of mouse to rat TNFα mRNA (Panel b) in transgenic and non-transgenic rat brain with or without ischemic brain injury. TNFα-transgenic (TNFα-Tg) and non-Tg rats underwent one hour of focal brain ischemia achieved by unilateral middle cerebral artery occlusion (MCAO). Tissue samples were obtained from the developing infarct core and the border zone approximating the ischemic penumbra in both groups of animals after 3 or 24 hours of post-ischemic reperfusion. As shown in Panel a, mean TNFα levels were significantly elevated within the border zone and infarct core after 24 hours of reperfusion in both TNFα-Tg and non-Tg rats, in comparison to animals of the respective type whether non-ischemic controls or ischemic rats sampled 3 hours after reversal of MCAO (n = 3 – 4 per group; p ≤ 0.001 for all pairwise comparisons; ANOVA/Fisher's test). The mean TNFα level in the border zone adjacent to the infarct core in the transgenic rat at 24 hours was significantly higher than the comparable level in the non-Tg animal (p ≤ 0.01; ANOVA/Fisher's test). Panel b of Figure 5 shows the mean ratios of mouse and rat TNFα mRNA corresponding to the TNFα protein levels from the same animals reported in Panel a. The mean ratio of mouse/rat TNFα mRNA was significantly higher in TNFα-Tg rats compared to the non-Tg animals only under control conditions without ischemia (p ≤ 0.01; ANOVA/Fisher's test). **p ≤ 0.01; ***p ≤ 0.001

To determine if ischemic stress altered the capacity for constitutive synthesis of TNFα protein in the brain of the transgenic animal, we examined regional levels of TNFα protein and mRNA in TNFα-Tg and non-Tg rat brain after 3 or 24 hours of post-ischemic reperfusion. Figure 7 is a composite bar graph showing mean levels of TNFα protein (Panel a) and the corresponding mean ratios of mouse to rat TNFα mRNA (Panel b) in the border zone approximating the ischemic penumbra and in the developing infarct core of both TNFα-Tg and non-Tg rats. The results in Panel a confirm that TNFα protein in control tissue from the transgenic rat is present in almost twice the quantity found in non-Tg littermates, as would be expected from assay of TNFα in non-ischemic brain tissue obtained from both animals shown in Figure 3. After 3 hours of reperfusion in animals subjected to MCAO, mean TNFα levels within the border zone and the developing infarct core in the TNFα-Tg rat rose above those in the non-Tg animal by statistically insignificant margins. However, mean TNFα levels became significantly elevated within the border zone and infarct core after 24 hours of reperfusion in both TNFα-Tg and non-Tg rats, in comparison to animals of the respective type whether non-ischemic controls or ischemic rats 3 hours after reversal of MCAO (n = 3 – 4 per group; p ≤ 0.001 for all pairwise comparisons; ANOVA/Fisher's test). In addition, the mean TNFα level in the border zone adjacent to the infarct core in the TNFα-Tg rat at 24 hours was significantly higher than the comparable level in the non-Tg animal (p ≤ 0.01; ANOVA/Fisher's test). Mean ratios of mouse and rat mRNA trended upward in all brain regions and at both sampling times in TNFα-Tg rats, compared to non-Tg littermates. However, this trend achieved statistical significance only between control animals that were not subjected to ischemic injury (p ≤ 0.01; ANOVA/Fisher's test).

**Figure 8:**
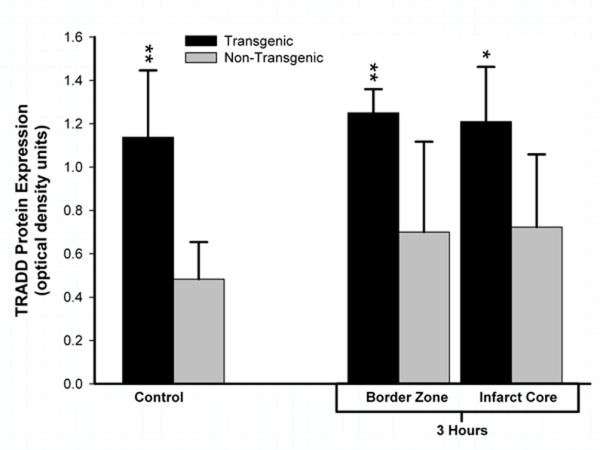
**Biological activity of TNFα in transgenic rat brain shown by TRADD protein expression**. This bar graph shows expression of TNF receptor-associated protein death domain (TRADD) protein in brain homogenates obtained from TNFα-transgenic (TNFα-Tg) and non-Tg rats. Samples were obtained after 3 hours of reperfusion following one hour of focal cerebral ischemia. TRADD protein expression is shown as mean ± SD optical density units measured from digitized electrophoretic bands. In ischemic TNFα-Tg brain, TRADD protein was expressed more intensely within the border zone approximating the ischemic penumbra (n = 5 per group; p ≤ 0.01; ANOVA/Fisher's test) and in the developing infarct core (p ≤ 0.05; ANOVA/Fisher's test) when compared to non-Tg littermates. TRADD was also elevated significantly in the normally-perfused hemisphere (control) of the TNFα-Tg rat compared to non-Tg animals (p ≤ 0.01; ANOVA/Fisher's test), reflecting increased constitutive expression of protein in response to interaction between active, soluble TNFα and its p55/TNFR1 receptor. There were no significant differences between TRADD protein expression in the normally-perfused hemisphere and the ischemic border zone or infarct core in TNFα-Tg rats (p = NS). *p ≤ 0.05; **p ≤ 0.01

To confirm that TNFα protein synthesized in the brain of the transgenic animal is biologically active, we quantified expression of TRADD protein. Homogenates of brain sampled from TNFα-Tg and non-Tg rats were incubated with a primary antibody that recognizes rat TRADD only after it has been activated through binding of TNFα to p55/TNFR1, thereby implying biological activity of the cytokine. Regional expression of TRADD protein was uniformly higher in TNFα-Tg rat brain than in non-Tg controls by statistically significant margins (see Figure 8). After 3 hours of post-ischemic reperfusion, mean TRADD expression in TNFα-Tg brain was increased in the infarct core (n = 5 per animal type; p ≤ 0.05; ANOVA/Fisher's test) and in the adjacent border zone approximating the ischemic penumbra (p ≤ 0.01; ANOVA/Fisher's test). To demonstrate that chronically elevated levels of TNFα enhanced expression of TRADD protein without ischemic stress, we sampled cortical tissue from the normally-perfused hemisphere in ischemic animals and found that TRADD was nearly threefold higher in the TNFα-Tg rat (p ≤ 0.01 compared to non-Tg animals; ANOVA/Fisher's test). There were no significant differences between TRADD protein expression in the normally-perfused hemisphere and the ischemic border zone or infarct core in the TNFα-Tg rat (p = NS).

### Apoptotic cell death after focal cerebral ischemia in the TNFα-Tg rat

**Figure 9:**
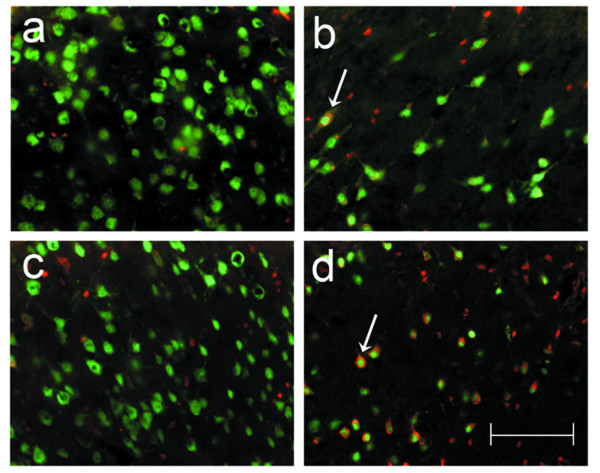
**Co-localization studies identifying neuronal apoptosis after focal cerebral ischemia**. A collage of 40× photomicrographs shows localization of activated caspase-3 (red) in NeuN-positive striatal neurons (green) after focal cerebral ischemia in TNFα-transgenic (TNFα-Tg) and non-Tg rats. Panels a and c are representative images of striatum in the left cerebral hemisphere of a non-Tg (a) and a TNFα-Tg (c) rat subjected to one hour of suture-occlusion of the right middle cerebral artery, followed by 24 hours of reperfusion. Note the absence of activated caspase-3 in NeuN-positive striatal neurons of the cerebral hemisphere contralateral to the ischemic injury in both animals. Panels b and d are taken from the ischemic striatum in the same non-Tg (b) and TNFα-Tg (d) rats. The presence of activated caspase-3 is clearly evident in NeuN-positive striatal neurons (arrows), many of which exhibit morphological changes consistent with ongoing cell death. Scale bar = 100 μm.

**Figure 10:**
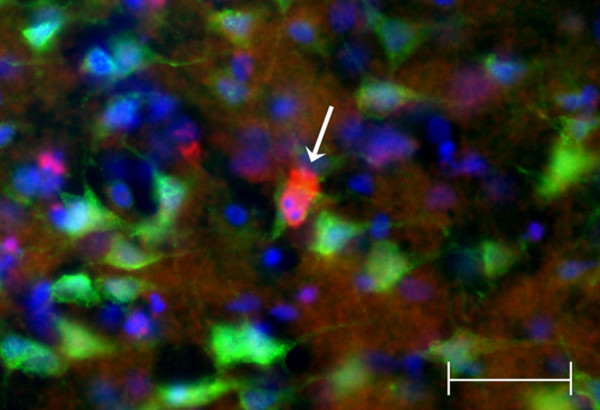
**Co-localization study revealing apoptotic bodies within degenerating neuron in ischemic TNFα-transgenic rat brain**. A single 40× photomicrograph shows localization of activated caspase-3 (red) in NeuN-positive cortical neurons (green) after focal cerebral ischemia in the TNFα-transgenic (TNFα-Tg) rat. The right cerebral cortex was sampled after one hour of suture occlusion of the ipsilateral middle cerebral artery, followed by 24 hours of reperfusion. In the representative NeuN-positive neuron identified by the arrow, there is caspase-3 activation with apoptotic bodies identified by Hoechst staining (blue). Scale bar = 50 μm.

We performed a qualitative assessment of apoptotic cell death, supported by quantitative assays of caspase-3 activation and DNA fragmentation in the ischemic brain of the TNFα-Tg and non-Tg rat. In Figure 9, co-localization studies in normal or ischemic striatum taken from transgenic and non-Tg animals prove that neurons show cytoplasmic caspase-3 activation while undergoing apoptotic death 24 hours after MCAO. Parallel studies performed to determine if caspase-3 activation was also observed in astroglia showed only rare astrocytes with co-localized GFAP and caspase-3 activation in ischemic striatum of both animals (data not shown). Comparison of Panel d to b in Figure 9 suggests, but does not prove, that degenerating neurons showing caspase-3 activation are more abundant in ischemic striatum within transgenic than in non-Tg brain. Apoptotic bodies were observed within degenerating neurons in ischemic TNFα-Tg rat brain (see Figure 10).

**Figure 11:**
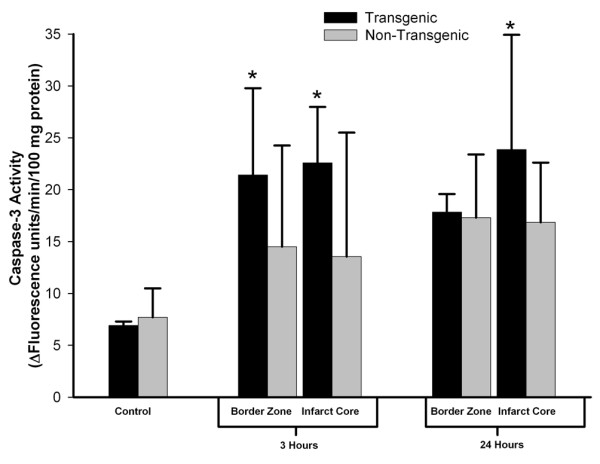
**Caspase-3 activity in TNFα-transgenic and non-transgenic rat brain after focal ischemia**. The bar graph shows caspase-3 activity in the brains of TNFα-transgenic (TNFα-Tg) and non-Tg rats (n = 3 – 4 per group and sampling time) with or without ischemic brain injury. TNFα-transgenic (TNFα-Tg) and non-Tg rats underwent one hour of focal brain ischemia achieved by unilateral middle cerebral artery occlusion. Tissue samples were obtained from the developing infarct core and the border zone approximating the ischemic penumbra in both groups of animals after 3 or 24 hours of post-ischemic reperfusion. Caspase-3 activity is presented as Δ fluorescence units/minute/100 mg protein (mean ± SD). Transgenic animals had significantly greater caspase-3 activity in the border zone region after 3 hours of reperfusion and in the infarct core at both 3 and 24 hours (p ≤ 0.05; ANOVA/Fisher's test).*p ≤ 0.05 compared to respective control.

**Figure 12:**
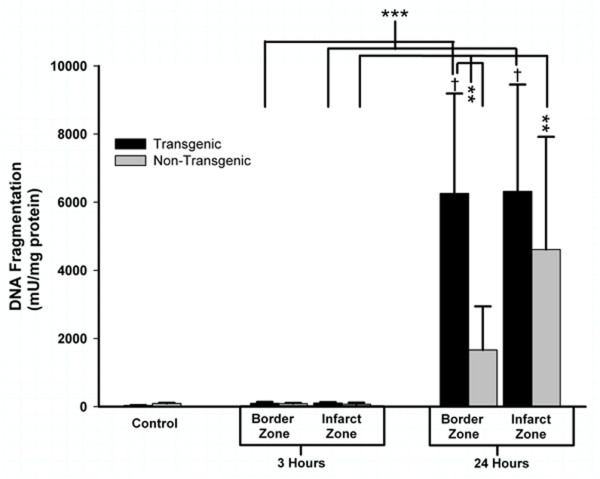
**Quantification of apoptotic cell death by DNA fragmentation in TNFα-transgenic and non-transgenic rat brain after focal ischemia**. The bar graph shows DNA fragmentation representing apoptotic cell death in brain sampled from TNFα-transgenic (TNFα-Tg) and non-Tg rats (n = 6 – 7 per group and sampling time) after one hour of unilateral middle cerebral artery occlusion (MCAO). Tissue samples were obtained from the developing infarct core and the border zone approximating the ischemic penumbra in both groups of animals after 3 or 24 hours of post-ischemic reperfusion. Neural cell death represented by DNA fragmentation is presented as mU/mg protein (mean ± SE). Cell death represented by DNA fragmentation was present at only negligible levels in non-ischemic TNFα-Tg and non-Tg controls (n = 12 per group) and after MCAO followed by 3 hours of post-ischemic reperfusion (p = NS for all pairwise comparisons). However, DNA fragmentation increased markedly after 24 hours of post-ischemic reperfusion in both TNFα-Tg and non-Tg rats. In the border zone approximating the ischemic penumbra in TNFα-Tg brain sampled at 24 hours, mean DNA fragmentation was increased by almost 200-fold compared to the respective non-ischemic control (p ≤ 0.0001; ANOVA/Fisher's test) and over 60-fold compared to border zone tissue sampled from TNFα-Tg brain at 3 hours (p ≤ 0.001; ANOVA/Fisher's test). DNA fragmentation in this region sampled from TNFα-Tg brain was almost 4-fold higher than that observed in non-Tg brain after 24 hours of post-ischemic reperfusion (p ≤ 0.01; ANOVA/Fisher's test). Within the infarct zone of TNFα-Tg brain sampled at 24 hours, mean DNA fragmentation was again increased by almost 200-fold compared to the respective non-ischemic control (p ≤ 0.0001; ANOVA/Fisher's test) and 60-fold compared to infarct zone tissue sampled from TNFα-Tg brain at 3 hours (p ≤ 0.001; ANOVA/Fisher's test). There was no significant difference observed between the elevated mean levels of DNA fragmentation within the infarct zone in TNFα-Tg and non-Tg rat brain after 24 hours of post-ischemic reperfusion (p = NS). **p ≤ 0.01; ***p ≤ 0.001; †p ≤ 0.0001

To pursue this observation implying that the TNFα-Tg rat may be susceptible to apoptotic neuronal death, we measured caspase-3 activity in the infarct core and in the border zone region approximating the ischemic penumbra in both animals. In the TNFα-Tg rat, caspase-3 activity was significantly greater than in non-Tg controls within the border zone and in the developing infarct after 3 hours of reperfusion (see Figure 11; n = 3 – 4 per group; p ≤ 0.05 for all pairwise comparisons; ANOVA/Fisher's test). At 24 hours, caspase-3 activity remained significantly higher within the infarct core of the TNFα-Tg rat compared to controls (p ≤ 0.05; ANOVA/Fisher's test). As a direct, quantitative measure of apoptotic cell death in ischemic brain, we assayed DNA fragmentation represented by histone-associated DNA complexes released into cellular cytoplasm. In the border zone approximating the ischemic penumbra in TNFα-Tg rat brain sampled at 24 hours, mean DNA fragmentation was increased by almost 200-fold compared to non-ischemic transgenic brain (see Figure 12; p ≤ 0.0001), over 60-fold compared to border zone tissue in ischemic TNFα-Tg brain sampled at 3 hours (p ≤ 0.001), and by 4-fold over that observed in non-Tg brain after 24 hours of post-ischemic reperfusion (p ≤ 0.01; n = 6 – 7 per group; all between-group comparisons by ANOVA/Fisher's test). Within the developing infarct core, DNA fragmentation was again increased by almost 200-fold compared to the non-ischemic transgenic brain (p ≤ 0.0001) and 60-fold compared to infarct zone tissue sampled from TNFα-Tg rat brain at 3 hours (p ≤ 0.001; all between-group comparisons by ANOVA/Fisher's test). There was no significant between-group difference in elevated mean levels of DNA fragmentation within the infarct core after 24 hours of post-ischemic reperfusion (p = NS). These quantitative results corroborate the findings of the co-localization studies in Figures 9 and 10 showing that the TNFα-transgenic rat is highly susceptible to apoptotic neuronal death after ischemia.

### Cerebral blood flow in the TNFα-Tg rat

**Figure 13:**
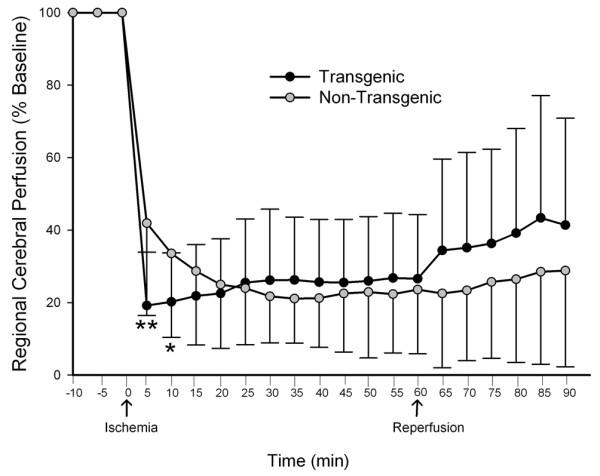
**Measurement of cortical perfusion during focal cerebral ischemia in TNFα-transgenic and non-transgenic rats**. The multi-line plot shows laser-Doppler flowmetry (LDF) measurements of cortical perfusion expressed as a percentage of pre-ischemic baseline in TNFα-transgenic (TNFα-Tg; n = 19) rats and non-Tg littermates (n = 21). Measurements were made every 5 minutes during 10 minutes of pre-ischemic baseline, 60 minutes of ischemia, and 30 minutes of post-ischemic reperfusion. Values were calculated as a percentage of pre-ischemic baseline and are presented as means ± SD. The TNFα-Tg rats had significantly lower mean LDF at 5 and 10 minutes immediately after the onset of ischemia (P = 0.0016 and 0.034, respectively; repeated measures ANOVA). *p ≤ 0.05; **p ≤ 0.01

After observing larger infarct volumes in the TNFα-Tg rat after 24 hours and 7 days of post-ischemic reperfusion, we were obligated to determine if the apparent susceptibility of the transgenic animal to ischemic injury was related to genetic modification of cerebral vascular reactivity or density. We used laser-Doppler flowmetry for serial measurement of cortical perfusion before, during, and immediately after MCAO to assess cerebral vascular reactivity in TNFα-Tg rats and non-Tg littermates. The multi-line plot in Figure 13 demonstrates that cortical perfusion within the infarct core of the TNFα-Tg rat fell below that of non-Tg controls shortly after MCAO. The TNFα-Tg rats had significantly lower mean LDF at 5 and 10 minutes of ischemia (n = 19 – 21 per animal type; p ≤ 0.01 and p ≤ 0.05, respectively; repeated measures ANOVA). There were no significant differences in LDF between the two groups of animals during the remaining 50 minutes of ischemia or 30 minutes of post-ischemic reperfusion.

**Figure 14:**
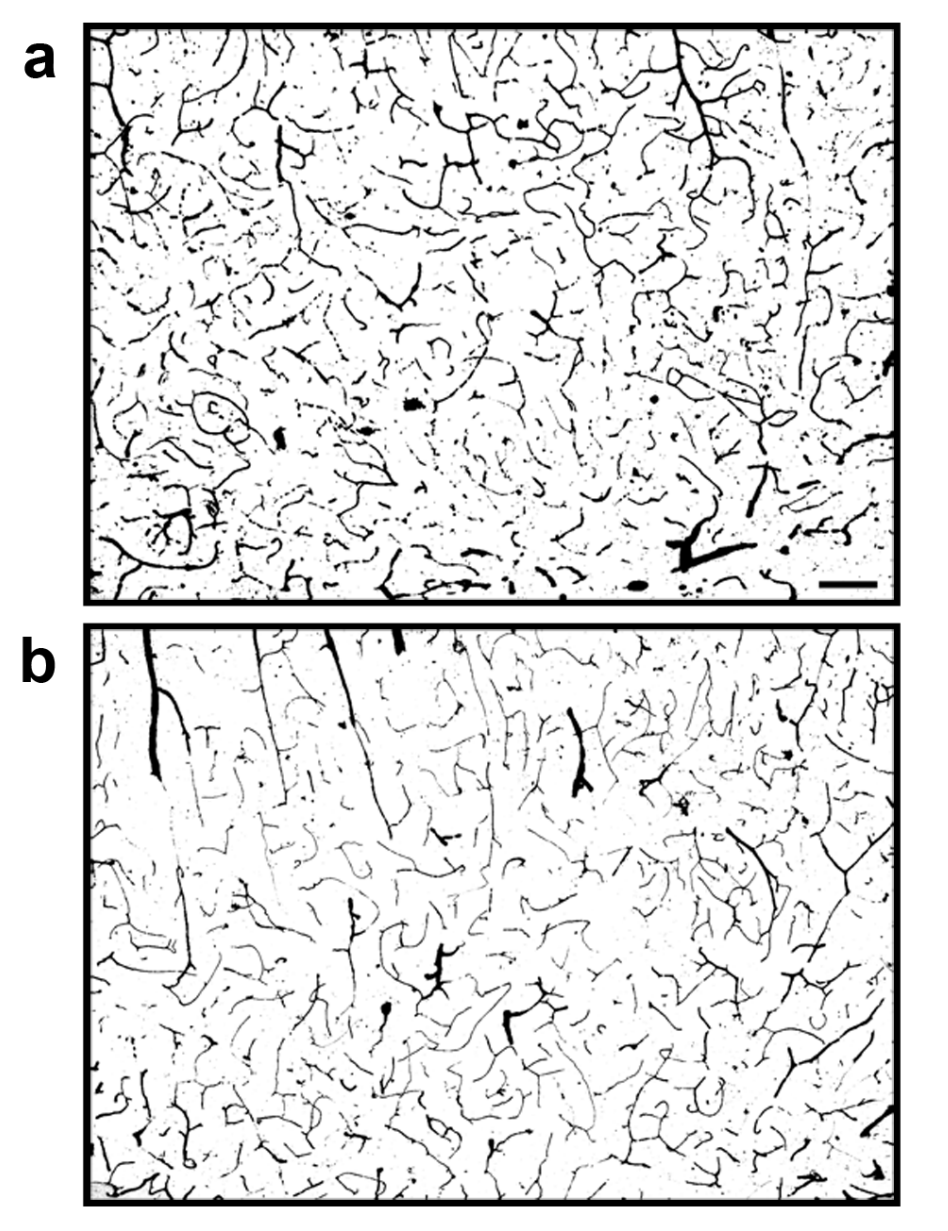
**Micro-vascular structures in the brains of TNFα-transgenic and non-transgenic rats**. These representative photomicrographs show ink-stained micro-vascular structures within the cortex of the TNFα-transgenic rat (Panel a) and a non-transgenic littermate (Panel b). There was no significant difference between samples in the number of micro-vascular structures quantified by stereology. Scale bar = 100 μm

Stereological analysis of vascular structures in the cortex showed that there was no change in micro-vascular anatomy caused by expression of the transgenic construct in the brain of the TNFα-Tg rat. The mean percent cortical area occupied by vascular structures in the cerebral cortex of the TNFα-Tg rats not subjected to MCAO was 6.20 ± 0.20, compared to 6.57 ± 0.31 in non-Tg littermates (p = NS). Figure 14 shows representative photomicrographs of micro-vascular structures within the cerebral cortex in both animal types.

## Discussion

This work confirms that the TNFα-Tg rat survives to adulthood, reproduces effectively, demonstrates upregulated expression of TNFα mRNA and protein in brain, and is susceptible to infarction by MCAO. Our results show that the high level of TNFα mRNA expressed constitutively in the brain of the transgenic animal is derived almost completely from the murine construct and not from the native rat gene. TNFα protein is increased selectively in the brain and by a statistically insignificant margin in the heart, kidney, and skeletal muscle, but remains low in serum and other external organs. TNFα-immunoreactive cells of neuronal morphology are present only in the neocortex of the transgenic animal. The distribution of the various cell populations in the brain of the TNFα-Tg rat appears no different than in the non-Tg animal, suggesting that the transgene has no measurable effect on the structural organization of the neocortex.

Our immunohistochemical studies indicate that the predominant cell type secreting TNFα in the brain of the transgenic rat is of neuronal lineage. This observation of constitutive neuronal expression differs importantly from previous studies showing that TNFα is frequently undetectable in quiescent neural cell populations, becoming upregulated in neurons and astroglial cells only after ischemic or post-traumatic stress [40,41]. In a comprehensive study of the evolution of TNFα expression in the brains of human patients with acute ischemic stroke, Sairanen and colleagues [6] found that TNFα expression appeared in neurons located within the infarct core and in the adjacent border zone during 24 hours after the onset of infarction. Reactive astrocytes then expressed TNFα immunoreactivity in both the injured hemisphere and in the contralateral hemisphere, beginning within 24 hours of stroke and persisting for up to 18 days afterward. These findings indicate that multiple neural cell types have the capacity to express TNFα, thereby emphasizing that this proinflammatory cytokine participates in an evolving brain injury response culminating in delayed involvement of "unaffected" tissue.

The suture-occlusion model of focal cerebral ischemia is feasible in the TNFα-Tg rat and produces a larger mean volume of infarction than in non-Tg littermates. We speculate that the greater infarct volume observed in the transgenic animal resulted from chronic overexpression of TNFα protein within the brain, causing receptor-mediated caspase activation that promoted neuronal apoptosis [42]. Binding of active, soluble TNFα to p55/TNFR1 will recruit TRADD, activate effector caspase-8, and cleave the executioner caspase-3 for activation [43]. Our observation that TRADD protein is expressed intensely within and outside of the ischemic focus in TNFα-Tg rat brain indicates that the cytokine produced in this animal is biologically active. We found significantly elevated levels of TNFα within ischemic tissue in the brain of the TNFα-Tg rat after 24 hours of post-ischemic reperfusion, but without corresponding increases in the component of mRNA transcribed from the murine TNFα gene. We speculate that the TNFα-Tg rat may have elevated TACE activity to facilitate synthesis of TNFα from its inactive precursor, thereby requiring no additional transcription of TNFα mRNA. Overall, our results suggest that it is soluble TNFα, not its membrane-bound precursor, which is active in the brain of the transgenic animal. Furthermore, our present findings allow us to conclude only that TNFα binds to p55/TNFR1 in the TNFα-Tg rat.

Our supposition that the pivotal mediator of increased lesion volume is the excess brain content of TNFα protein in the transgenic rat is corroborated by the work of Barone and colleagues [13]. To directly examine the role of early TNFα production in the evolution of secondary ischemic injury, these investigators administered exogenous TNFα by intracerebroventricular (ICV) injection 24 hours before MCAO to achieve permanent or transient focal ischemia in rats. They found that motor function, hemispheric edema, and infarct volume were affected adversely in a dose-dependent manner by TNFα. Furthermore, the specificity of the effect of higher doses of TNFα to cause progressively larger infarcts was confirmed by reduction of infarct volume after ICV injections of an anti-TNFα neutralization antibody or of soluble TNFR1 before and after MCAO. This relationship between lesion volume and ambient level of TNFα has been confirmed repeatedly in animal models of focal cerebral ischemia [14,15,44,45] and has even been observed in human stroke victims. Montaner and co-workers [46] found positive correlations between elevated blood levels of TNFα or interleukin-6 and the volume of hypoperfused brain observed after bolus-tracking perfusion MRI studies in stroke patients.

We examined brain perfusion by LDF to determine if the larger mean infarct volume in the TNFα-Tg rat was caused by an unanticipated effect of the constitutively expressed TNFα mRNA or translated protein on the cerebral circulation. We found that cortical perfusion within the infarct core in the brain of the TNFα-Tg rat fell to 20% of pre-ischemic baseline within 5 minutes after MCAO. Beyond the first 10 minutes of MCAO, there was no significant difference in cortical flow between transgenic and non-Tg animals for the remaining 50 minutes of focal ischemia and 30 minutes of post-ischemic reperfusion. This result suggests that constitutive upregulation of TNFα protein could accentuate brain response to focal circulatory arrest and may have contributed to the larger mean percent infarct volume observed in transgenic animals. However, the transience of the effect on LDF in the ischemic TNFα-Tg rat is self-evident and was not corroborated by underlying changes in micro-vascular anatomy, as was shown in our stereological analysis of cortical vascular density.

Our speculation of a link between elevated TNFα in brain and cortical perfusion is corroborated by Lavine and colleagues [45] who found that pre-ischemic administration of an anti-TNFα neutralization antibody one hour before MCAO significantly improved perfusion in the infarct core and in the peri-infarct region after reversal of MCAO in non-Tg animals. Alternately, Savitz and others [47] showed that pre-ischemic administration of carvedilol, a mixed α and β adrenoreceptor antagonist, reduced TNFα mRNA copy number, infarct volume, and ischemic apoptosis after MCAO but had no effect on LDF. Recent studies suggest that TNFα may affect brain endothelial cell function or microvascular integrity, even if its influence on regulation of cerebral hemodynamic function after ischemia remains ambivalent. Kimura and coworkers [48] reported that TNFα induced cleavage of caspase-3 and promoted apoptosis in cultured brain microvascular endothelial cells. Hosomi and others [44] found that TNFα may increase microvascular permeability after ischemia by upregulating matrix metalloproteinases (MMPs). They observed that intravenous infusion of an anti-TNFα neutralization antibody immediately after reversal of MCAO inhibited upregulation of MMP-9 and membrane type 1-MMP, thereby lowering infarct volume and attenuating ischemic brain edema.

We have confidence that the unique cellular anatomy and the response to ischemic brain injury observed in our transgenic animal cannot be attributed to random genetic heterogeneity. Transgenic animals are often produced on a hybrid background and then backcrossed to optimize genetic homogeneity. The TNFα-Tg rats were produced on the outbred S-D rat strain. All subsequent generations of transgenic animals have been prepared by backcrossing onto this same strain. We have used non-Tg littermates as controls in all the assays we performed to quantify TNFα mRNA and protein levels and verify cellular anatomy. TNFα-Tg animals express a uniform phenotype that differs consistently from the non-Tg littermates. Therefore, we believe that the unique features of the TNFα-Tg rat must be attributed to segregation of the transgene allele.

With its chronic upregulation of TNFα in brain, the TNFα-Tg rat is a clinically relevant model of secondary inflammatory changes after cerebral infarction in humans. Although non-Tg animals show increased levels of endogenous TNFα in response to ischemia, the transgenic rat has greater research potential because of the ease with which signal transduction and pro-apoptotic pathways may be studied under conditions of prolonged activation *in vivo*. Even though TNFα-Tg mice share this asset, their role in the assessment of experimental therapeutic agents will be limited by small size, restricted vascular access, and constrained use in functional assessment of motor and cognitive behavior. The TNFα-Tg rat is a valuable new tool for the study of cytokine-mediated signal transduction paths in ischemic brain injury.

## Competing interests

David S. Grass, Ph.D., is a salaried employee of Xenogen Biosciences, Cranbury, New Jersey, USA. Xenogen Biosciences provided no financial support for the submitted work, will not realize financial gain from publication of the work, and did not pay the article-processing charge. All other authors declare that they have no financial or non-financial competing interests.

## Authors' contributions

LCP conceived of the study, supervised its design and coordination, and wrote the manuscript. MSK obtained the transgenic animal described in the study and contributed to the manuscript. SS performed quantitative stereology and contributed to the manuscript. JES performed histological studies and contributed to the manuscript. RJK performed statistical analyses and contributed to the manuscript. YL conducted preliminary studies with the transgenic animal. DSG provided the transgenic animal used in the study and contributed to the manuscript. All authors read and approved the final manuscript.

## References

[B1] Emsley HC, Tyrrell PJ (2002). Inflammation and infection in clinical stroke. J Cereb Blood Flow Metab.

[B2] Mayne M, Ni W, Yan HJ, Xue M, Johnston JB, Del Bigio MR, Peeling J, Power C (2001). Antisense oligodeoxynucleotide inhibition of tumor necrosis factor-alpha expression is neuroprotective after intracerebral hemorrhage. Stroke.

[B3] Tomimoto H, Ihara M, Wakita H, Ohtani R, Lin JX, Akiguchi I, Kinoshita M, Shibasaki H (2003). Chronic cerebral hypoperfusion induces white matter lesions and loss of oligodendroglia with DNA fragmentation in the rat. Acta Neuropathol (Berlin).

[B4] Lotocki G, Alonso OF, Dietrich WD, Keane RW (2004). Tumor necrosis factor receptor 1 and its signaling intermediates are recruited to lipid rafts in the traumatized brain. J Neurosci.

[B5] Tomimoto H, Akiguchi I, Wakita H, Kinoshita A, Ikemoto A, Nakamura S, Kimura J (1996). Glial expression of cytokines in the brains of cerebrovascular disease patients. Acta Neuropathol (Berlin).

[B6] Sairanen T, Carpén O, Karjalainen-Lindsberg ML, Paetau A, Turpeinen U, Kaste M, Lindsberg PJ (2001). Evolution of cerebral tumor necrosis factor-alpha production during human ischemic stroke. Stroke.

[B7] Carlstedt F, Lind L, Lindahl B (1997). Proinflammatory cytokines, measured in a mixed population on arrival in the emergency department, are related to mortality and severity of disease. J Intern Med.

[B8] Vila N, Castillo J, Dávalos A, Chamorro A (2000). Proinflammatory cytokines and early neurological worsening in ischemic stroke. Stroke.

[B9] Zaremba J, Skrobanski P, Losy J (2001). Tumor necrosis factor-alpha is increased in the cerebrospinal fluid and serum of ischaemic stroke patients and correlates with the volume of evolving brain infarct. Biomed Pharmacother.

[B10] Sairanen TR, Lindsberg PJ, Brenner M, Carpén O, Sirén AL (2001). Differential cellular expression of tumor necrosis factor-α and Type I tumor necrosis factor receptor after transient global forebrain ischemia. J Neurol Sci.

[B11] Saito K, Suyama K, Nishida K, Sei Y, Basile AS (1996). Early increases in TNF-α, IL-6, and IL-1β levels following transient cerebral ischemia in gerbil brain. Neurosci Lett.

[B12] Liu Y, Jacobowitz DM, Barone F, McCarron R, Spatz M, Feuerstein G, Hallenbeck JM, Siren AL (1994). Quantitation of perivascular monocytes and macrophages around cerebral blood vessels of hypertensive and aged rats. J Cereb Blood Flow Metab.

[B13] Barone FC, Arvin B, White RF, Miller A, Webb CL, Willette RN, Lysko PG, Feuerstein GZ (1997). Tumor necrosis factor-alpha: a mediator of focal ischemic brain injury. Stroke.

[B14] Dawson DA, Martin D, Hallenbeck JM (1996). Inhibition of tumor necrosis factor-alpha reduces focal cerebral ischemic injury in the spontaneously hypertensive rat. Neurosci Lett.

[B15] Nawashiro H, Martin D, Hallenbeck JM (1997). Inhibition of tumor necrosis factor and amelioration of brain infarction in mice. J Cereb Blood Flow Metab.

[B16] Nawashiro H, Tasaki K, Ruetzler CA, Hallenbeck JM (1997). TNF-alpha pretreatment induces protective effects against focal cerebral ischemia in mice. J Cereb Blood Flow Metab.

[B17] Sharif SF, Hariri RJ, Chang VA, Barie PS, Wang RS, Ghajar BG (1993). Human astrocyte production of tumor necrosis factor-α, interleukin-1β and interleukin-6 following exposure to lipopolysaccharide endotoxin. Neurol Res.

[B18] Sawada M, Kondo N, Suzumura A, Marunouchi T (1989). Production of tumor necrosis factor-α by microglia and astrocytes in culture. Brain Res.

[B19] Wajant H, Pfizenmaier K, Scheurich P (2003). Tumor necrosis factor signaling. Cell Death Differ.

[B20] Bruce AJ, Boling W, Kindy MS, Peschon J, Kraemer PJ, Carpenter MK, Holtsberg FW, Mattson MP (1996). Altered neuronal and microglial responses to excitotoxic and ischemic brain injury in mice lacking TNF receptors. Nat Med.

[B21] Gary DS, Bruce-Keller AJ, Kindy MS, Mattson MP (1998). Ischemic and excitotoxic brain injury is enhanced in mice lacking the p55 tumor necrosis factor receptor. J Cereb Blood Flow Metab.

[B22] Probert L, Akassoglou K, Pasparakis M, Kontogeorgos G, Kollias G (1995). Spontaneous inflammatory demyelinating disease in transgenic mice showing central nervous system-specific expression of tumor necrosis factor. Proc Natl Acad Sci USA.

[B23] Kollias G, Hurst J, deBoer E, Grosveld F (1987). The human beta-globin gene contains a downstream developmental specific enhancer. Nucleic Acid Res.

[B24] Brinster RL, Chen HY, Trumbauer ME, Yagle MK, Palmiter RD (1985). Factors affecting the efficiency of introducing foreign DNA into mice by microinjecting eggs. Proc Natl Acad Sci USA.

[B25] Holtz ML, Craddock S, Pettigrew LC (2001). Rapid expression of neuronal and inducible nitric oxide synthases during post-ischemic reperfusion in rat brain. Brain Res.

[B26] Minger SL, Geddes JW, Holtz ML, Craddock SD, Whiteheart SW, Siman RG, Pettigrew LC (1998). Glutamate receptor antagonists inhibit calpain-mediated cytoskeletal proteolysis in focal cerebral ischemia. Brain Res.

[B27] Pettigrew LC, Holtz ML, Craddock SD, Minger SL, Hall N, Geddes JW (1996). Microtubular proteolysis in focal cerebral ischemia. J Cereb Blood Flow Metab.

[B28] Longa EZ, Weinstein PR, Carlson S, Cummins R (1989). Reversible middle cerebral artery occlusion without craniotomy in rats. Stroke.

[B29] Dubal DB, Kashon ML, Pettigrew LC, Ren JM, Finklestein SP, Rau SW, Wise PM (1998). Estradiol protects against ischemic injury. J Cereb Blood Flow Metab.

[B30] Belayev L, Alonso OF, Busto R, Zhao W, Ginsberg MD (1996). Middle cerebral artery occlusion in the rat by intraluminal suture. Neurological and pathological evaluation of an improved model. Stroke.

[B31] Bederson JB, Pitts LH, Germano SM, Nishimura MC, Davis RL, Bartkowski HM (1986). Evaluation of 2,3,5-triphenlytetrazolium chloride as a stain for detection and quantification of experimental cerebral infarction in rats. Stroke.

[B32] Swanson RA, Morton MT, Tsao-Wu G, Savalos RA, Davidson C, Sharp FR (1990). A semiautomated method for measuring brain infarct volume. J Cereb Blood Flow Metab.

[B33] McEwen ML, Springer JE (2005). A mapping study of caspase-3 activation following acute spinal cord contusion in rats. J Histochem Cytochem.

[B34] Shimizu H, Ohgoh M, Ikeda M, Nishizawa Y, Ogura H (2007). Caspase-3-like protease activity-independent apoptosis at the onset of neuronal cell death in the gerbil hippocampus after global ischemia. Biol Pharm Bull.

[B35] Cholet N, Seylaz J, Lacombe P, Bonvento G (1997). Local uncoupling of the cerebrovascular and metabolic responses to somatosensory stimulation after neuronal nitric oxide synthase inhibition. J Cereb Blood Flow Metab.

[B36] Scheff SW, Bernardo LS, Cotman CW (1978). Decrease in adrenergic axon sprouting in the senescent rat. Science.

[B37] Paxinos G, Watson C (1998). The rat brain in stereotaxic coordinates.

[B38] Mouton PR (2002). Principles and practices of unbiased stereology: an introduction for bioscientists.

[B39] Allan SM, Rothwell NJ (2001). Cytokines and acute neurodegeneration. Nat Rev Neurosci.

[B40] Feuerstein GZ, Wang X, Barone FC (1998). The role of cytokines in the neuropathology of stroke and neurotrauma. Neuroimmunomodulation.

[B41] Harrington JF, Messier AA, Levine A, Szmydynger-Chodobska J, Chodobski A (2005). Shedding of tumor necrosis factor type 1 receptor after experimental spinal cord injury. J Neurotrauma.

[B42] Martin-Villalba A, Herr I, Jeremias I, Hahne M, Brandt R, Vogel J, Schenkel J, Herdegen T, Debatin KM (1999). CD95 ligand (Fas-L/APO-1L) and tumor necrosis factor-related apoptosis-inducing ligand mediate ischemia-induced apoptosis in neurons. J Neurosci.

[B43] Muzio M, Salvesen GS, Dixit VM (1997). FLICE induced apoptosis in a cell-free system. Cleavage of caspase zymogens. J Biol Chem.

[B44] Hosomi N, Ban CR, Naya T, Takahashi T, Guo P, Song XY, Kohno M (2005). Tumor necrosis factor-alpha neutralization reduced cerebral edema through inhibition of matrix metalloproteinase production after transient focal cerebral ischemia. J Cereb Blood Flow Metab.

[B45] Lavine SD, Hofman FM, Zlokovic BV (1998). Circulating antibody against tumor necrosis factor-alpha protects rat brain from reperfusion injury. J Cereb Blood Flow Metab.

[B46] Montaner J, Rovira A, Molina CA, Arenillas JF, Ribo M, Chacon P, Monasterio J, Alvarez-Sabin J (2003). Plasmatic level of neuroinflammatory markers predict the extent of diffusion-weighted image lesions in hyperacute stroke. J Cereb Blood Flow Metab.

[B47] Savitz SI, Erhardt JA, Anthony JV, Gupta G, Li X, Barone FC, Rosenbaum DM (2000). The novel beta-blocker, carvedilol, provides neuroprotection in transient focal stroke. J Cereb Blood Flow Metab.

[B48] Kimura H, Gules I, Meguro T, Zhang JH (2003). Cytotoxicity of cytokines in cerebral microvascular endothelial cell. Brain Res.

